# The Record of Yellow Fever in the United States for the Past Does Not Sustain the Mosquito Theory*Read before the Brazos Valley Medical Association at Bryan, Texas, November, 13, 1901.

**Published:** 1902-01

**Authors:** J. P. Oliver

**Affiliations:** Caldwell, Texas


### TEXAS MEDICAL JOURNAL.

ESTABLISHED JULY, 1885.

PUBLISHED MONTHLY.—SUBSCRIPTION $1.00 A YEAR.

VOL. XVII. AUSTIN, JANUARY, 1902. No. 7.

### Original Contributions.

For Texas Medical Journal.

### The Record of Yellow Fever in the United States for the Past Does Not Sustain the Mosquito Theory.*

BY J. P. OLIVER, M. D., CALDWELL, TEXAS.

Perhaps the most marvelous announcement of the new century was made at the Pan-American Medical Congress in Havana, Cuba, in February, last, by Drs. Reed, Carroll and Agramonte, that yellow fever is solely transmitted by the mosquito known as the *culex faciatus.* I shall endeavor to show that the record of yellow fever, from its earliest recognition to the last epidemic in the United States, does not sustain this scientific theory.

According to the best data at my command, the first trace of yellow fever was observed about the end of the fifteenth century, or the beginning of the sixteenth, at San Domingo and Porto Rico, in the West Indies. Columbus, landing at the former place in 1493, lost the greater number of his men within a year after their arrival from a disease described as being yellow as saffron, or gold. From 1544 to 1635, when it appeared at Guadalupe, there is no record of any outbreak. But thenceforward it occurred at irregu-

*Read before the Brazos Valley Medical Association at Bryan, Texas, November, 13, 1901.

lar intervals. In the seventeenth century it spread along the east coast of America as far as 8° south latitude and 42° north latitude, appearing for the first time in Boston, Mass., in 1693. In the eighteenth century it appeared on the west coast of South America and extended to Europe and Madagascar. Commercial and military activities doubtless favored its spread and increased its frequency of epidemics, eighteen having been recorded in San Domingo within the century. Early in 1700 it reached New York. At the beginning of the nineteenth century it reached 47° north latitude in America, and also prevailed in the Canary Islands, in Leghorn and in the maritime cities of Spain and Portugal. In 1853, 1867, 1873, 1878, 1897-8-9, it spread to a number of cities, towns and villages in the interior and as far north as the town of Gallipolis, in Ohio, of the American continent, being transported by railroads and steam vessels, establishing the fact, beyond a reasonable doubt, its infection by fomites and, in some instances, by a recrudescence or reinfection, and the *culex faciatus* was never thought of until the experiments in Cuba.

During this century it seems to have relinquished its hold upon the northern cities of the Atlantic coast and has been concentrated chiefly along the southern borders of the Atlantic and Gulf coasts. Yellow fever has prevailed almost annually as an endemic or epidemic at Havana, Cuba, from April to December for perhaps the last two centuries, and from time immemorial it has been endemic at Vera Cruz. It is endemic in the West Indies, and is rarely, if ever, absent, until the United States took possession of Cuba and put Havana in a better sanitary condition than it has been perhaps for the last one hundred years. The seasons of the year have but little influence in eradicating it, though the disease is most fatal from May to August, the temperature and moisture being favorable for its development, as it seems to spread in a temperature above 70°. Since the disease is dependent in a great measure upon an elevated temperature, its occurrence is limited to tropical and subtropical regions, or to countries having a tropical climate, or a summer of sufficient length, heat and moisture. Yellow fever has seldom, if ever, shown itself except in maritime regions, elevated but a few feet above the sea. From Brazil to Vera Cruz in one direction, and from Barbadoes to Tampico in the other. On the Atlantic coasts it has extended as far north as Boston, Mass., and Portland, Me., while in the Mississippi Valley it has appeared three times at high as Memphis, Tenn., north 35°. In an eastern direction, but within the same parallels, it has extended to Cadiz, Heres, Carthegena, Malaga, Alicant, Seeville, Barcelona and other cities on the coast and in the interior of Spain. It has prevailed several times at Gibraltar, once at Rockford, twice at Lisbon, and once at Leghorn. It prevailed at Monte Vidio; latitude 34° 50′ south in 1859 and in 1872; in Buenos Ayres in 1859 and 1871, once at Panama and Callao, and at Lima and Cuzeo in Peru in 1854 and in 1856. In America it reached from latitude 36° south to 45° north on the Atlantic coast, and to 31° north in the Mississippi Valley. On the Pacific coast it extends from 15° south to 9° north. Longitude limits in the same country 60° to 97° west. In the Eastern Hemisphere it occurs within the limits of 42° north and 8° south. It is said not to appear in the East Indies or in China. It has been said that yellow fever never extended above 2500 feet above the sea. This has been shown to be incorrect, for it has prevailed in the elevated table lands of Caracas 3000 feet above the sea level on more than one occasion. In 1854 and 1856 it committed fearful ravages in Cuzco at an elevation of 11,378 feet, and other places in the Andes at 14,000 feet. Great variety in this respect exists, for at Jalapa, in Mexico, in the same parallel with Vera Cruz, 4300 above the sea, it has never prevailed as an epidemic. The hills in Jamaica and San Domingo are free from the pestilence, which rages in its low lands.

Dr. S. H. Green, city physician of Boston, Mass., several years since, expressed the opinion that the chances are that yellow fever will not reach Boston. He adds, however, that it is a mistaken idea that the fever cannot rage there if once introduced, and cites the prevalence in this city of the scourge in 1639. It was called the "fleet fever," having been brought to that port by infected seamen of the British fleet. These are said to have been the first instances of yellow fever in this country. The scourge, as far as statistics show, broke out in the West Indies in the early part of the seventeenth century, and these islands have constituted its favorite haunts. In Europe it has invaded Spain, Portugal, Italy, France and Belgium. In Africa it has appeared along the south and west coasts, and in South America it has prevailed in British Guinea, Colombia, Peru, Bolivia, Buenos Ayres and the Brazils. In North America it has invaded Honduras and Mexico, and in the following twenty-four States of the Union: Massachusetts, Rhode Island, New Hampshire, Connecticut, New Jersey, Pennsylvania, New York, Delaware, Maryland, Illinois, Missouri, Ohio, Kentucky, Virginia, North Carolina, South Carolina, Georgia, Alabama, Tennessee, Mississippi, Arkansas, Louisiana, Florida and Texas. It has never made its appearance on the great continent of Asia, nor in any islands of the Pacific coast of the American continent.

Yellow fever has invaded present territorial limits of the United States in eighty-eight different years—1669, 1693, 1702, 1705, 1713, 1728, 1732, 1739, 1741, 1744, 1745, 1747, 1748, 1762, 1765, 1791, 1792, 1793, 1794, 1795, 1796, 1797, 1798, 1799, 1800, and all the years of this century so far with the exception of 1836, 1840, 1850, 1860, 1861, 1866 and 1874. In seventy more out of the eighty-eight years there is ample evidence of importation, and in seventy-one out of these seventy-seven the evidence points to the West Indies as the source of infection. The fever has never acquired a domicile in this country, although some authors believe it to be of local origin. The deaths in this country in 1878 amounted to 20,000, three-fifths of whom were minors, and the loss to New Orleans alone commercially has been computed at $15,335,000.

Dr. Green was one of the board of experts authorized by Congress to investigate the yellow fever epidemic of 1878, and his opinions are well worthy of notice. For the instructive facts following, I am indebted to the special report of the fevers of New Orleans, particularly the yellow fever of 1849, by Professor E. D. Fenner, M. D., of New Orleans. The bare mention of this distinguished and lamented physician and thorough gentleman is introduction enough. Dr. Fenner writes: "It appears from the records that a period of nine years from the first of January, 1841, to the first of January, 1850, there was 4133 cases of yellow fever in the Charity Hospital of New Orleans. A supplementary report by Dr. Simons gives 13,000, showing almost conclusively that the fever was imported annually to the city of New Orleans by commercial intercourse from infected cities. In 1853, during the epidemic of yellow fever in New Orleans, Columbia, a little town where the author read medicine, situated on the west bank of the Ouachita river at the foot of the hills between four and five hundred miles above New Orleans, was the head of navigation on that river not only for North Louisiana, but for Arkansas also. Merchandise of nearly every variety was stored in this little town. Some time in August, if my memory serves me right, yellow fever developed, and became epidemic in this little town, causing the death of nearly one-half of its population before it was stopped by cold weather. Will any one deny the fact of importation here from New Orleans, or will they assume that the *culex faciatus* was imported along with the goods stored in this little town, and perchance got in his work.

Again in 1867, when yellow fever was prevailing at New Orleans and other places on the Gulf and Atlantic coasts and places in the interior, the first case that occurred in Grenada, Miss., was traced to the house of a lady who had ordered a silk dress through the mail from New Orleans. Fumigation was not practiced then as now. But let us come closer. "Keating's History of the Epidemic of 1878," in speaking of Camp Joe Williams, which was located near Memphis, Tenn., and was peopled by a thousand or more refugees from the city, says a number of persons who had become infected before leaving the city developed the disease after reaching the camp, but from such cases there was no spread. Surgeon R. B. Nall, who was in charge of the camp, says: "The remarkable and favorable feature of Camp Joe Williams was that the disease did not spread among its inhabitants. Nor did those who visited the camp from the surrounding country contract the disease. On the arrival of the refugees every article of bedding and clothing which favored the propagation of the disease was destroyed by fire. Five of the eight male nurses employed in the hospital, after nursing for three or four weeks fifteen or twenty patients in all stages of the fever, thinking themselves proof against the disease, went to the city to offer themselves to the Howard Association as nurses, because of the higher prices paid. They found the sick bountifully supplied with nurses from elsewhere, and were unable to obtain positions, and returned to camp. Four of these men died in the hospital in which they had nursed; the fifth was found dead between the city and the camp. The other three nurses did not visit the city, but remained in the hospital during the epidemic seventy-two days, nursed and buried their comrades, but were not attacked themselves." Dr. John H. Purnell, State health officer of Mississippi, who had charge of the yellow fever epidemics at Edwards in 1897 and Jackson in 1898, says the foregoing is a plain unembellished statement of a reputable physician, and contains several points of interest in connection with the recent investigations carried on in Cuba. If the bedding and clothing in the hospital at Camp Joe Williams during the summer weather did not become infected, is there not good reason to suppose that the material taken from Las Animas Hospital and Quemados in November and December escaped likewise. The non-immune physicians and nurses who attended yellow fever patients in 1899 failed to develop the disease while there, but did develop it afterwards from an infected center. (Information from Surgeon H. Carter.) All bedding and clothing coming from the infected city was burned, and while yellow fever cases developed in those who had become infected before leaving the city, none occurred in any others. Is it not reasonable to suppose that the precautionary measures were responsible for the immunity, since it is known that mosquitos plentifully abound around the suburbs as well as in the city of Memphis?

In the epidemic of yellow fever at Edwards, Miss., there is every indication that the fever was imported from Ocean Springs by a lady who had visited her daughter at that place and returned to her home at or near Edwards on or about August 8, 1877. At the time of her visit a disease was prevailing the nature of which had not been determined, but shortly afterwards was declared to be yellow fever. From this case a population of about 925 people, 325 whites and 600 colored, within sixty days after the first case had occurred, 299 whites and 427 negroes had developed yellow fever. If the mosquito was the important factor in disseminating the fever among these people, they certainly did their work well and rapidly.

In the epidemic of yellow fever in 1878, extending from the Gulf of Mexico throughout the Mississippi Valley to Gallipolis, Ohio, and resulting in 72,000 cases with 16,000 deaths, is there any reasonable doubt to suppose that the mosquito disseminated the germs? It seems to me that there is abundant evidence to show that yellow fever is not only propagated by fomites, but is recrudescent, as instanced at Memphis one epidemic following another.

Dr. Purnell reports another incident in point. On the 14th of September, 1897, Mr. and Mrs. B. refugeed to their home some miles from Edwards, Miss. A number of people were in their house at the time of their arrival, but all left with the exception of Mr. and Mrs. H. (daughter and son-in-law of Mr. and Mrs. B.), and three servants. Mr. B. developed yellow fever on September 15th and died on the 17th. He was buried on the same day, and everything was destroyed that had been in the room. The room was disinfected and sealed up. The portion of the house in which the case occurred was separated from the other rooms by a twelve foot hall, in which sulphur was kept burning for two weeks. Mrs. B. had all of her clothing destroyed with the exception of a cashmere shawl. This she was desirous of keeping, and it was placed in an upstairs room close to the eaves. On October 5th, thinking all danger of a recurrence of the disease passed, preparations were made for the return of the family. Mr. H. visited the upstairs room, and finding the shawl, threw it down stairs and called to some one to remove it out doors. His wife responded. As she started out she met her mother, who attempted to rescue her property. Further than this I was not told, but two days after the episode Mrs. H. developed yellow fever, followed by Mr. H. one day later, and Mrs. B. developed a case on the fourth day. Ten days subsequently two of the servants suffered attacks, presumably from a second infection of the house; perhaps by the removal of the shawl.

In Dr. Matas' article in the October (1897) issue of the *New Orleans Medical and Surgical Journal,* he quotes an illustration of conveyance by fomites. The facts, he says, were furnished by Dr. Shannon, of Ocean Springs, Miss., in a letter to Dr. Bemis. On the 14th of October, 1883, Major J. B. D. died of yellow fever in Ocean Springs. I moved the family to a healthy locality, when you saw Miss B. not allowing them to take any articles from the rooms where the husband and father had died. The children applied to me for a lock of their father's hair, which I refused, but the older daughter, now dead, prevailed upon the nurse to give it to her. She placed it in an old envelope that had been torn open at the end and carefully folded the torn end down, thus practically sealing it, and laid it away among other old letters. On the 4th of November, 12:30 p. m., she brought this envelope out upon the gallery and opened it for the first time to examine the lock of hair, and to show it to her aunt, Miss S., who was visiting her, and upon inhaling the concentrated poison confined in the envelope and emanating from the hair, she exclaimed, "O, what a peculiar smell." She handed the envelope to Miss S., who, unconscious of danger, also inhaled the messenger of death with a similar exclamation, when Mrs. B., who was standing near by, reached her hand for the letter, but was prevented by a child who wanted to be taken up in her arms. This gave time for reflection, and she admonished the young ladies of the possible danger. The envelope was carefully folded and with its fatal contents replaced in the drawer where it has been since the 14th of October. This drawer has been almost daily opened. On the following Saturday, November 10th, at 9 p. m., Miss S. was taken sick with a chill, and Miss B. about 2 a. m., some five hours later, the period being less than seven days in both cases. No other person handled the fatal envelope or in any way came in contact with it, and there is, after the most careful inquiry, no suspicion of any other source of infection in those two cases. Miss S. died on November 14th, and Miss B. on November 16th, twenty-seven days between the death of a single case of yellow fever and the development of a second case. It is presumable that in the removal from the house that the mosquitos were left behind. If, however, one or two had been carried from the house it would have been a strange coincidence, indeed, for them to have selected these two young ladies, who had been in contact with the hair, for victims.

In "Keating's History of the Yellow Fever Epidemic of 1878," quite a number of cases are reported from infection by fomites, to which you can refer for further information along this line. Many instances of ships plying between infected ports and others not infected have caused the disease after being long out from the infected port. It would seem that it has been established, at least by the Cuban commission, that the *culex faciatus* is a disseminator of the germs of yellow fever. But it appears to me that this commission proves too much. First, the houses built were perhaps in as good a sanitary condition as they could possibly be made under the circumstances. Second, the investigation was made in a country where yellow fever is and has been endemic perhaps for centuries. Third, it was made at the season of the year most favorable for its non-development. Fourth, the length of time required to determine infection of the mosquito, twelve days being required. Fifth, none but the female propagates the infecting material. There are about two hundred species of mosquitos. Each female mosquito is said to lay about two hundred eggs at a time.

Again, I quote from Dr. Waldaer, Ph. G., M. D., of Shreveport, La., who was in both epidemics of yellow fever in Mississippi in 1897-8. At Nitta Yuma, Miss., in 1897, Mr. C., who attended Mr. C. H.'s funeral at Edwards, Miss., returned to his home and had yellow fever. Mr. B. and others who visited him during his illness contracted the disease. A cordon was established around the town, except on the west side, where the railroad track was made the dividing line. A strict quarantine was maintained until November 11th. The mosquito was there in all of his glory, yet not a case made its appearance outside the line. During the epidemic of yellow fever of 1878 in Vicksburg, Miss., there was a strict quarantine kept at the county jail. As a result not a case of yellow fever made its appearance within its walls, and the mosquito was permitted to go and come unmolested.

While in conversation with Dr. G., of Greenville, Miss., he related the following as to the spread of yellow fever at Stoneville, Miss. Mrs. Q., of Stoneville, was in labor, and sent to Greenville for consultation. Dr. G. answred the call. Before his arrival the lady had been delivered of a boy. Dr. G. remained three or four hours to rest his horses, and returned home. Mrs. Q. two days later had a chill in the night, and in less than a week died of black vomit of yellow fever. Dr. G. was convinced that he carried the fomites to Mrs. Q., who developed yellow fever, as the fever was at Greenville, but undetermined. Mrs. Q. had the first case in Stoneville.

The quarantine instructions of Chief Surgeon Howard of Havana, having the endorsement of Surgeon General Sternberg, contained the following: "Medical officers taking care of yellow fever patients need not be isolated. They can attend other patients and associate with non-immunes with perfect safety to the garrison. Nurses and attendants taking care of yellow fever patients should be isolated so as to avoid any possible danger of their conveying mosquitos to non-immunes." Why the nurses are more liable to convey mosquitos to non-immunes than the physicians I can not understand.

In conclusion, permit me to say that I have no objection to the war being waged to exterminate the mosquito, for in the destruction of his habitat it only improves sanitation. Clean up our cities, especially the coast cities and villages, and keep them clean. Observe proper quarantine systems of fumigation and other means, and I am sure this will be the best protection not only of the citizens of our coast cities, but of commerce also. Every one who has studied the history of yellow fever in New Orleans knows full well that after General Butler took possession of New Orleans in 1862 and placed it in a good sanitary condition, not only has the mortality of yellow fever been greatly reduced, but other infectious diseases also. During the last epidemic of yellow fever there the mortality was very little over 10 per cent. This speaks volumes for good sanitary and fumigating laws in every city on the Atlantic and Gulf coasts. It will be a sad travesty not only on the State governments, but the United States also, if an efficient quarantine system is ever relinquished to the fallacy of the mosquito theory. Talk about shotgun quarantine—it will be in full force then; the people are the government in this country. I am in favor of a health officer in the cabinet of the President of the United States, as much so as I am in favor of a secretary of war or any other cabinet officer. The commerce of the United States is a big thing, but the health and lives of 75,000,000 people are far more valuable than this universal commercialism and expansion that is being foisted on this government. Finally, when the Isthmian canal is built, somewhere on the Gulf coast of Texas a great commercial city will be erected, surpassing perhaps Greater New York. Ships from every nation will come for Texas commerce. Infectious diseases of every character will be brought to our shores, when the intelligence and ingenuity of the best men of the country will be brought into requisition. But Texas undivided will be the grandest in the whole galaxy of States.

